# Simulated evolution applied to study the genetic code optimality using a model of codon reassignments

**DOI:** 10.1186/1471-2105-12-56

**Published:** 2011-02-21

**Authors:** José Santos, Ángel Monteagudo

**Affiliations:** 1 Department of Computer Science, University of A Coruña, Campus de Elviña s/n, 15071 A Coruña, Spain

## Abstract

**Background:**

As the canonical code is not universal, different theories about its origin and organization have appeared. The optimization or level of adaptation of the canonical genetic code was measured taking into account the harmful consequences resulting from point mutations leading to the replacement of one amino acid for another. There are two basic theories to measure the level of optimization: the statistical approach, which compares the canonical genetic code with many randomly generated alternative ones, and the engineering approach, which compares the canonical code with the best possible alternative.

**Results:**

Here we used a genetic algorithm to search for better adapted hypothetical codes and as a method to guess the difficulty in finding such alternative codes, allowing to clearly situate the canonical code in the fitness landscape. This novel proposal of the use of evolutionary computing provides a new perspective in the open debate between the use of the statistical approach, which postulates that the genetic code conserves amino acid properties far better than expected from a random code, and the engineering approach, which tends to indicate that the canonical genetic code is still far from optimal. We used two models of hypothetical codes: one that reflects the known examples of codon reassignment and the model most used in the two approaches which reflects the current genetic code translation table. Although the standard code is far from a possible optimum considering both models, when the more realistic model of the codon reassignments was used, the evolutionary algorithm had more difficulty to overcome the efficiency of the canonical genetic code.

**Conclusions:**

Simulated evolution clearly reveals that the canonical genetic code is far from optimal regarding its optimization. Nevertheless, the efficiency of the canonical code increases when mistranslations are taken into account with the two models, as indicated by the fact that the best possible codes show the patterns of the standard genetic code. Our results are in accordance with the postulates of the engineering approach and indicate that the main arguments of the statistical approach are not enough to its assertion of the extreme efficiency of the canonical genetic code.

## Background

The canonical genetic code is not universal although it is present in most complex genomes. Its establishment is still under discussion once the discovery of non-standard genetic codes altered the "frozen accident" [1]. Woese [2] was one of the first to consider the adaptability of the genetic code. He suggested that the patterns within the standard genetic code reflect the physicochemical properties of amino acids. An argument in favor is the fact that in the canonical genetic code the amino acids with similar chemical properties are coded by similar codons.

There are three basic theories on the origin of the organization of the genetic code [3]. The stereochemical theory claims that the origin of the genetic code must lie in the stereochemical interactions between anticodons or codons and amino acids. The second one is the physicochemical theory, which claims that the force defining the origin of the genetic code structure was the one that tended to reduce the deleterious effects of physicochemical distances between amino acids codified by codons differing in one base. The third one is the coevolution hypothesis [4,5], which suggests that the structure of the genetic code reflects the biosynthetic pathways of amino acids through time and the error minimization at the protein level is just a consequence of this process. This coevolution theory suggests that codons, originally assigned to prebiotic precursor amino acids, were progressively assigned to new amino acids derived from the precursors as biosynthetic pathways evolved. For other authors as Higgs [6], the driving force during the build-up of the standard code is not the minimization of the effects of translational error, and the main factor that influenced the positions in which new amino acids were added is that there should be minimal disruption of the protein sequences that were already encoded. Nevertheless, the code that results is one in which the translational error is minimized.

Several previous works have studied the genetic code optimality. Most authors have quantified the efficiency of a possible code taking into account the possible errors in the codon bases. Generally, a measurement of changes in a basic property of the codified amino acids was used considering all the possible mutations in a generated code. The most efficient code is one that minimizes the effects of mutations, as this minimization implies a smaller phenotypic change in the codified proteins.

Once the efficiency of a code has been measured, different criteria are used to assess whether the genetic code is in some sense optimal. These analyses fall into two main classes: statistical [7] and engineering [8]. The first one considers the probability of random codes more efficient than the standard genetic code. With this alternative for measuring code optimality, the standard genetic code is compared with many randomly generated alternative codes. These considerations define the so-called "statistical approach" [7]. Comparing the error values of the standard genetic code and alternative codes indicates, according to the authors using this approach [9-13], the role of selection. The main conclusion of these authors is that the genetic code conserves amino acid properties far better than expected from a random code.

In a first computational experiment with this alternative, Haig and Hurst [12] corroborated that the canonical code is optimized to a certain extent. They found that of 10,000 randomly generated codes, only two performed better at minimizing the effects of errors, when polar requirement [2] was taken as the amino acid property, concluding that the canonical code was a product of natural selection for load minimization. Freeland and Hurst [9] investigated the effect of weighting transition errors differently from transversion errors and the effect of weighting each base differently, depending on reported mistranslation biases. When they used weightings to allow for biases in translation, they found that only one in every million randomly generated alternative codes was more efficient than the standard genetic code.

With a similar methodology, Gilis et al. [14] took into account the frequency at which different amino acids occur in proteins and found that the fraction of random codes that beat the canonical code decreases. Torabi et al. [15] considered both relative frequencies of amino acids and relative gene copy frequencies of tRNAs in genomic sequences which were used as estimates of the tRNA content [16]. Zhu et al. [17] included codon usage differences between species and Marquez et al. [18] tested the idea that organisms optimize their codon usage as well as their genetic code: codons with lower error values might be used in preference to those with higher error values, to reduce the overall probability of errors, although their conclusions were the opposite.

Sammet et al. [19], using a genotype-to-phenotype mapping based on a quantitative model of protein folding, compared the standard genetic code to seven of its naturally occurring variants with respect to the fitness loss associated to mistranslation and mutation. According to the authors' methodology, most of the alternative genetic codes were found to be disadvantageous to the standard code, that is, the standard code is generally better able to reduce the translation load than the naturally occurring variants.

The second alternative for measuring code optimality is the so-called "engineering approach", followed, for example, by Di Giulio [8,20]. The approach uses a "percentage distance minimization" (p.d.m.) which compares the standard genetic code with the best possible alternative. The p.d.m. determines code optimality on a linear scale, as it is calculated as the percentage in which the canonical genetic code is in relation to the randomized mean code and the most optimized code. Therefore, it is defined as *(∆_mean _- ∆_code_)/(∆_mean _- ∆_low_)*, where *∆_mean _*is the average error value, obtained by averaging over many random codes, and *∆_low _*is the best (or approximated) *∆ *value. This approach tends to indicate that the genetic code is still far from optimal.

With this methodology, Di Giulio [21] estimated that the standard genetic code has achieved 68% minimization of polarity distance, by comparing the standard code with random codes that reflect the structure of the canonical code and with the best code that the author obtained by a simulated annealing technique. The author indicates that the minimization percentage can be interpreted as the optimization level reached during genetic code evolution. With this methodology, the authors in [22] also considered the evolution of the code under the coevolution theory. We previously analyzed the evolution of codes within the coevolution theory [23].

We used the mean square (MS) measurement [9,12] (Methods Section) to quantify the relative efficiency of any given code. We considered two possibilities to generate alternative codes: the first one is the model of hypothetical codes that reflects the current genetic code translation table (model 1), which is most used in the literature. Two restrictions were considered [9,12]:

1. The codon space (the 64 codons) was divided into the 21 nonoverlapping sets of codons observed in the standard genetic code, each set comprising all codons specifying a particular amino acid in the standard code.

2. Each alternative code is formed by randomly assigning each of the 20 amino acids to one of these sets. The three stop codons remain invariant in position, these being the same stop codons of the standard code.

This choice of a small part of the vast space of possible codes, with these conservative restrictions, as Novozhilov et al. [24] indicate, "is based on the notion that the block structure of the standard code is a consequence of the structure of the complex between the cognate tRNA and the codon in mRNA where the third base of the codon plays a minimum role as a specificity determinant".

As the codon set structure of the standard genetic code is unchanged, only considering permutations of the amino acids coded in the 20 sets, there are 20! (2.43·10^18^) possible hypothetical codes. Without restrictions in the mapping of the 64 codons to the 21 labels there would be more than 1.51·10^84 ^general codes [25].

In this work we considered the commented restrictive codes. Nevertheless, as Higgs [6] indicates, none of the known examples of codon reassignment occurs by swapping the amino acids assigned to two codon blocks. Instead, one or more codons assigned to one amino acid are reassigned to another, so one block of codons decreases in size while the other increases. Furthermore, the amino acid that acquires the codon is almost always a neighbor of the one that loses it. As Higgs [6] states, "The reason for this is that reassignments of codons to neighbouring amino acids can be done by changing only a single base in the tRNA anticodon". Hence, we also studied a second alternative with these possible restricted hypothetical codes which consider these codon reassignments (model 2), model not considered in the previous literature.

## Methods

The optimality of a code is related to its relative efficiency when different errors are considered in the DNA sequence or in the transcription and translation machinery of the protein synthesis. The efficiency generally considers these possible errors to take into account the possible changes in codified amino acids and their properties [7-18,20-27]. A code which, on average, generates fewer changes is more efficient, as the effects of errors are minimized.

### Encoding and genetic operators

**Figure 1:**
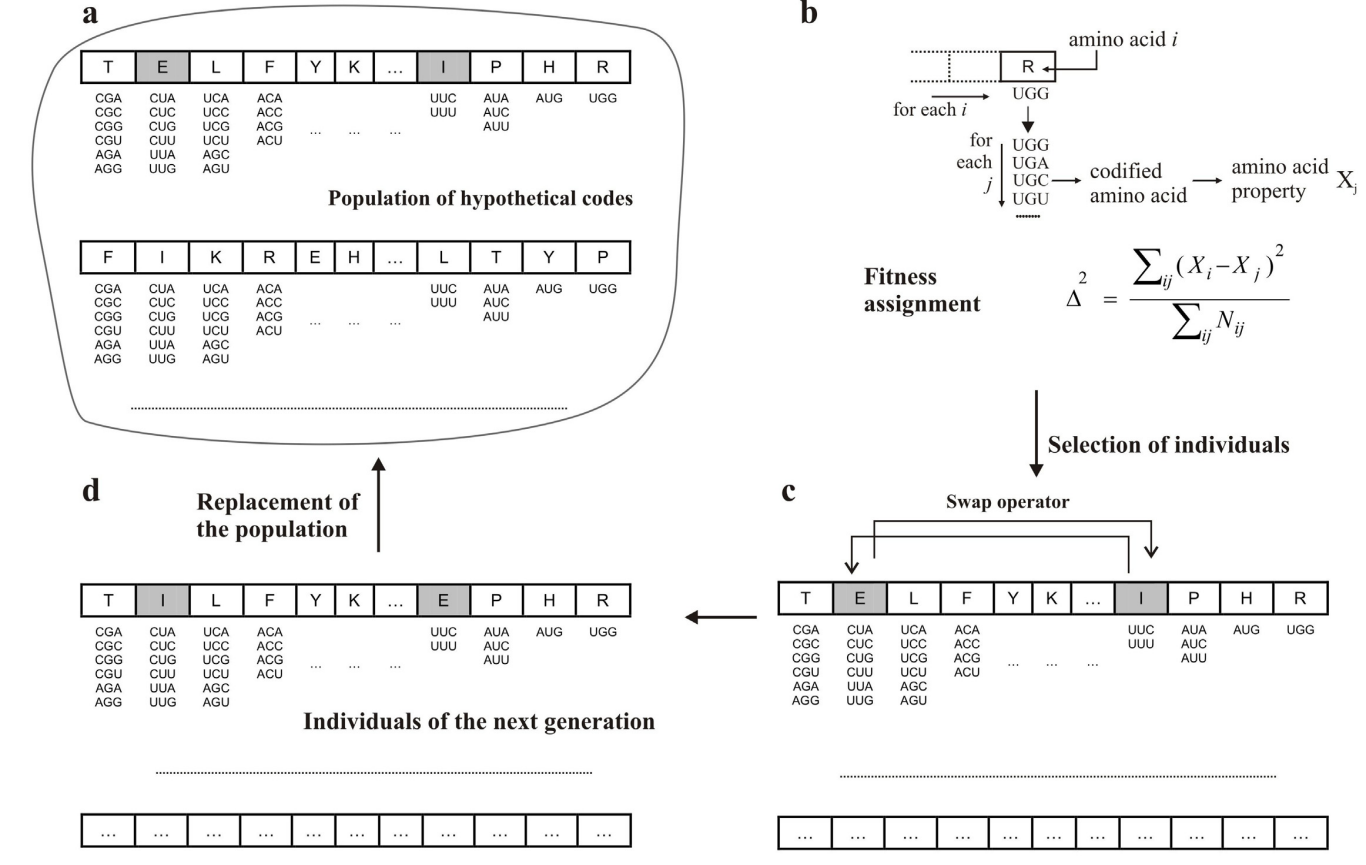
**Evolutionary computing methodology applied to the search for better adapted codes**. (a) Each individual of a genetic population encodes a possible hypothetical code, which is evaluated according to its efficiency to changes or mutations in the codon bases. (b) This efficiency defines the fitness of each individual, which is used by the genetic algorithm to select the individuals (c) in which a genetic operator (swap) is applied to define the individuals of the next generation (model 1 of hypothetical codes). (d) This evolutionary process is iterated through generations.

An adapted genetic algorithm (GA) [28,29] was used to search for alternative codes that were more optimized than the standard genetic code. Each individual of the genetic population must encode a hypothetical code. Model 1 of alternative codes considered permutations of the amino acids coded in the 20 codon sets observed in the canonical code, so each individual has 20 positions, and each position encodes the particular amino acid associated with the codon set (Figure 1). The use of a simple algorithm ensures that the individuals of the initial population encode the 20 amino acids. Three codons are used for the stop label, which are the same as those of the canonical code.

In model 1, the GA used a swap operator. The operator interchanges the contents of two codon sets, that is, once two positions are randomly selected, the amino acids codified by the two respective codon sets are swapped. Figure 1 shows how this operator works.

In model 2 of hypothetical codes each individual has 64 positions, corresponding to the 64 codons. In each hypothetical code, 3 codons are reserved for the stop signal. In this case, the genetic operator models the known codon reassignments [6]. This operator can be summarized as follows:

1. Choose a random codon from the 61 codons that encode an amino acid.

2. The encoded amino acid is copied (duplicated) in another codon (randomly chosen) which differs only in one letter with respect to the first codon. If the amino acid to replace is the only instance in the hypothetical code, then the operator is not applied.

In both models, tournament was used as selection operator. It chooses the best in a window of randomly selected individuals from the population. Hence, the size of the window determines the required selective pressure. Moreover, elitism of the best individual was used, that is, this individual is kept in the next generation without changes.

### Fitness function in the Genetic Algorithm

The fitness function was the measurement that calculates the mean square (MS) change in an amino acid property resulting from all possible changes to each base of all the codons within a given code [9,12]. Any one change is calculated as the squared difference between the property value of the amino acid coded for by the original codon and the value of the amino acid coded for by the new (mutated) codon. As most authors [9,12,20-22] we used the polar requirement as the amino acid property. This property can be considered as a measurement of hydrophobicity and it was introduced by Woese as a measurement for the polarity of an amino acid, which is defined as a partitioning coefficient of an amino acid in a water/pyrimidine system [2]. The final error is an average of the effects of all the substitutions over the whole code. Hence, the error ∆ is defined as:

$$\Delta^{2}=\frac{\sum_{ij}{\left(X_{i}-X_{j}\right)}^{2}}{\sum_{ij}N_{ij}}$$

where *N_ij _*is the number of times the *i-th *amino acid changes into the *j-th *amino acid, and *X_i _*is the value of the amino acid property of the *i-th *amino acid. The changes from and to "stop" codons are ignored, while synonymous changes (the mutated codon encoding the same amino acid) are included in the calculation. The MS value defines the fitness value of a given code and the evolutionary algorithm will try to minimize it.

## Results and discussion

**Figure 2:**
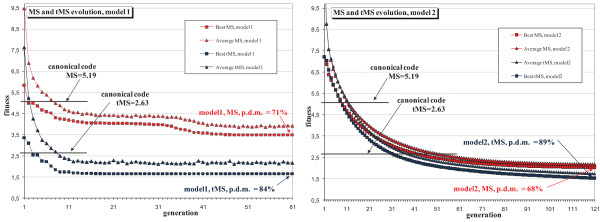
**Evolutions of alternative codes with MS and tMS**. Evolution of the best individual and the average quality of the population using MS and tMS as fitness with codes that reflect the canonical code translation table (model 1) and with the model of natural codon reassignments (model 2). MS is the average mean square error of the effects of possible changes in the three codon bases. tMS incorporates the quantification of mistranslation in each particular base. These curves of fitness evolution were the result of an average of 50 evolutions with different initial populations. The horizontal lines indicate the corresponding MS or tMS values of the canonical code.

We tested the implemented GA, searching for alternative codes, with the two definitions of models of hypothetical codes previously explained. Figure 2 shows the evolution of the MS across the generations of the genetic algorithm. The quality (fitness) of the best individual and the average quality of the population were the result of an average of 50 evolutions with different initial populations. The population size was 1,000 individuals for the different tests and we used tournament selection with a size of 3% of the population. The selected individuals pass to the next generation, applying the suitable genetic operators for each model (Methods Section).

The mean value of the best final codes was 3.506 using model 1, with a low standard deviation of 0.031. The best value found by Freeland and Hurst [9] was 4.7 and the MS value of the standard genetic code is 5.19. The p.d.m., using the best value obtained by the GA, was 71% with these restrictive codes. Figure 2 shows that evolution easily finds better adapted codes, although the p.d.m. value shows good adaptability of the standard genetic code. The p.d.m. with the codes of model 2 was 68%, this value being lower since the freer evolution of codes can obtain better optimal codes.

We repeated the analysis taking into account the errors as a function of the base position in the codon. Table 1 shows the quantification of mistranslation used in [9] as well as in this work to weight the relative efficiency of the three bases. It presents a summary of the empirical data on the frequency of transition and transversion mutations at the three codon positions. The MS is changed to tMS, which weights the errors according to the values shown in Table 1.

**Table 1:** Quantification of mistranslation used to weight the relative efficiency of the three bases in the tMS calculation.

Combined weighting	First base	Second base	Third base
For transitions	1	0.5	1

For transversions	0.5	0.1	1

Using model 1, there was an increase from a p.d.m. value of 71% in the MS case to a p.d.m. value of 84% when the mistranslation biases were considered in the fitness calculation. Using model 2, the increase was larger, from a p.d.m. value of 68% in the MS case to a value of 89% using tMS. This implies that the standard code is better adapted when we consider the quantification of mistranslations. This agrees with the results obtained in the statistical study of Freeland and Hurst [9] (these authors used only model 1). Note that using the two fitness functions, MS and tMS, model 2 obtains better values, although using tMS the GA needs more generations to overcome the corresponding values found with model 1, so the evolutions with model 2 are shown with more generations. The reason of the better values with model 2 is that, with the movements of this model, there is the possibility to reach the codes obtained with model 1, so the GA has a larger landscape where to find better codes.

The evolution of the quality curves leads to the same conclusion: Evolution requires more generations to obtain a better individual with a better value than that of the canonical code when using tMS. This is clearer with the known codon reassignments model. With the average quality we have the same effect, as the GA has greater difficulty in obtaining better individuals than the canonical code.

**Figure 3:**
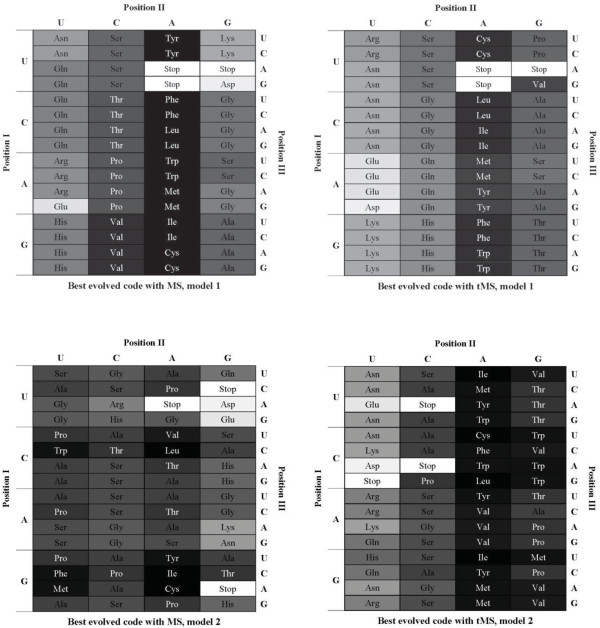
**Tables of best evolved codes with MS and tMS in restrictive codes (model 1), and best evolved codes in the codon reassignments model (model 2)**. The position of each codified amino acid is shaded by a gray scale, representing its polar requirement value.

Figure 3, with the usual representation of the genetic code, corresponds with the assignments of best evolved codes using MS and tMS in the restrictive codes as well as with the model of codon reassignments. The position of each codified amino acid is shaded by a gray scale representing its polar requirement value. Although there are very different assignments of amino acids with respect to the canonical code, the two alternative restrictive codes present two patterns that are correlated with systematic errors in the processes of replication and translation, which are also present in the standard genetic code [30]. Pattern I: Amino acids are more similar to each other along the first codon position than they are along the second. This "column-like" pattern corresponds to the high rate of translational misreading in the first codon position; Pattern II: Along the second position, amino acids associated with pyrimidine bases (U,C) or purine bases (A,G) are more similar within these sets than between them. This is associated with mutational bias in replication, in which transitions (mutations within these base sets) occur more frequently than transversions (mutations of a base in one set to a base in the other set). Pattern I is present in all the evolved codes except for the evolved code using model 2 and MS, where is more difficult to recognize such pattern. Pattern II is clearer in the best codes with tMS, especially with model 2 of hypothetical codes. This is logical because the tMS variant models the different frequency of transition and transversion mutations.

**Figure 4:**
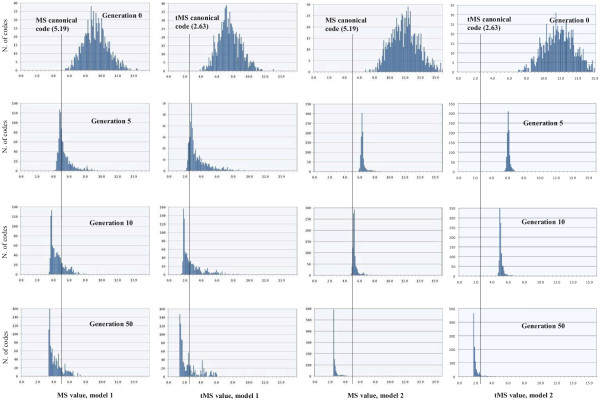
**Histograms of different generations of evolutionary runs with both models of hypothetical codes**. The histograms correspond to the initial generation, generations 5 and 10, and generation 50 when there is not significant improvement in the evolutionary processes. In the histograms, the *x*-axis gives a particular range of categories of MS/tMS values whereas the *y*-axis gives the number of individuals with an MS/tMS in that category. The vertical lines in the histograms indicate the category into which the MS/tMS value of the canonical code falls.

The MS or tMS values of each sample of codes in each generation form a probability distribution against which the standard genetic code MS or tMS values may be compared. Figure 4 shows the histograms at four stages of the evolutionary processes: initial population, generations 5, 10 and 50. The histograms of the initial populations present a similar distribution as the ones of Freeland and Hurst [9], as the populations are random. A better code (better than the canonical code) was not found by chance in those initial populations. At the end of the evolutionary processes, the situation changed radically, where most of the individuals showed a better MS/tMS than that of the standard genetic code.

## Conclusions

We used a genetic algorithm to search for better adapted hypothetical codes and as a method to guess the difficulty in finding such alternative codes, allowing to clearly situate the canonical code in the fitness landscape. We are emphasizing what simulated evolution search can provide about such difficulty of discovering possible better codes than the canonical one, and we must take into account that our methodology does not provide possible evolutionary pathways by which the canonical code reached its current state, as done by other authors [6].

From our GA simulations we can infer several conclusions. First, our results are not in disagreement with the main result of the statistical approach, as it is shown in the histograms of the initial populations, because such distributions of codes demonstrate, using the MS and tMS cost functions, that the canonical code is much better than random codes. Moreover, we agree with Knight et al. [26] when they state that the code could be trapped in a local, rather than global, optimum, and when they indicate that the average effect of amino acid changes in proteins is unlikely to be perfectly recaptured by a single linear scale of physical properties [26]. Nevertheless, with the information provided by the evolution of the histograms (Figure 4), now we do not agree with the authors who focus their analyses on the statistical approach [7,9-11,27] when they favor it because, as they emphasize, the approach takes into consideration that the possible random codes form a Gaussian distribution of error values [13]. According to the authors, the canonical genetic code is "extremely efficient" [9]. When they used an amino acid similarity based on the PAM 74-100 matrix, Freeland et al. [27] stated "if theoretically possible code structures are limited to reflect plausible biological constraints, and amino acid similarity is quantified using empirical data of substitution frequencies, the canonical code is at or very close to a global optimum for error minimization" [27]. Nevertheless, Di Giulio has questioned this work, as the title of his article "the origins of the genetic code cannot be studied using measurements based on the PAM matrix because this matrix reflects the code itself, making any such analysis tautologous" clearly explains [31].

However, regarding the comments of the authors focused on the statistical approach, even beginning with the Gaussian distributions of random codes in the initial genetic populations, the GA simulations indicate that it is very easy to improve the adaptability level of the standard genetic code. The better codes were obtained with low selective pressure and in few generations. Hence, the canonical code is clearly far from optimal, as also revealed by the position of the optimality values of the canonical code in the curves of quality evolution (Figure 2) for the two models considered. In this sense, we agree with the engineering approach as this alternative tends to indicate that the canonical code is still far from optimal. Nevertheless, the more realistic model of the known codon reassignments shows a slightly better efficiency of the canonical code with respect to the first model, as revealed by the greater difficulty of the GA to overcome the optimality value of the canonical code, as Figures 2 and 4 indicate.

## Authors' contributions

JS planned the studies and wrote the manuscript. AM performed the different experiments. Both authors discussed the results and implications and commented on the manuscript at all stages. Both authors read and approved the final manuscript.
